# Culture-Based Wastewater Surveillance for the Detection and Monitoring of Antimicrobial Resistance in Staphylococcal Species

**DOI:** 10.3390/vetsci13010014

**Published:** 2025-12-23

**Authors:** Douha Shouqair, Rashed Alghafri, Mohammed Naji, Abdulla Albastaki, Rania Nassar, Lobna Mohamed, Bisola Aloba, Bayan S. Awad, Fatima Al Dhaheri, Dean Everett, Ihab Habib, Mahmood Almashadani, Ahmed A. Shibl, Jorge Rodríguez, Danesh Moradigaravand, Stefan Monecke, Ralf Ehricht, Mushtaq Khan, Richard Goering, Abiola Senok

**Affiliations:** 1 College of Medicine, Mohammed Bin Rashid University of Medicine and Health Sciences, Dubai P.O. Box 505055, United Arab Emirates; 2 General Department of Forensic Science and Criminology, Dubai Police, Dubai P.O. Box 1493, United Arab Emirates; 3 Department of Pediatrics, College of Medicine and Health Sciences, United Arab Emirates University, Al Ain P.O. Box 15551, United Arab Emirates; 4 Department of Public Health and Epidemiology, College of Medicine and Health Sciences, Khalifa University, Abu Dhabi P.O. Box 127788, United Arab Emirates; 5 Infection Research Unit, Khalifa University, Abu Dhabi P.O. Box 127788, United Arab Emirates; 6 Department of Veterinary Medicine, College of Agriculture and Veterinary Medicine, United Arab Emirates University, Al Ain P.O. Box 15551, United Arab Emirates; 7 Public Health Research Center, New York University Abu Dhabi, Abu Dhabi P.O. Box 129188, United Arab Emirates; 8 Department of Chemical Engineering and P. Engineering, Khalifa University, Abu Dhabi P.O. Box 127788, United Arab Emirates; 9 Biological and Environmental Science and Engineering Division, King Abdullah University of Science and Technology, Thuwal 23955-6900, Saudi Arabia; 10 Leibniz Institute of Photonic Technology (IPHT), Leibniz Center for Photonics in Infection Research (LPI), 07745 Jena, Germany; 11 InfectoGnostics Research Campus, 07743 Jena, Germany; 12 Center for Translational Medicine (CETRAMED), Jena University Hospital, Friedrich Schiller University Jena, 07747 Jena, Germany; 13 Institute of Physical Chemistry, Friedrich-Schiller University, 07743 Jena, Germany; 14 Department of Medical Microbiology and Immunology, College of Medicine and Health Sciences, United Arab Emirates University, Al Ain P.O. Box 15551, United Arab Emirates; 15 Zayed Bin Sultan Center for Health Sciences, United Arab Emirates University, Al Ain P.O. Box 17666, United Arab Emirates; 16 Department of Medical Microbiology and Immunology, Creighton University School of Medicine, Omaha, NE 68178, USA; 17 School of Dentistry, Cardiff University, Cardiff CF14 4XY, UK

**Keywords:** *Staphylococcus*, wastewater-based surveillance, antimicrobial resistance, wastewater treatment plants

## Abstract

Wastewater-based surveillance (WBS) is valuable for monitoring antimicrobial resistance (AMR). Staphylococci are key targets, as wastewater can facilitate gene transfer and resistance emergence. Data on WBS for population-level AMR in the Arabian-Gulf remain limited. This study assessed *Staphylococcus* diversity and resistance in Dubai wastewater. Samples were collected over eight months from nine community sites, two hospital nodes, and two wastewater treatment plants (WWTPs) and were analysed by culture-based method. Ninety-six *Staphylococcus* isolates were recovered from community, hospital, and WWTP influent, with no growth in effluent. Most isolates (n/N = 88/96) were *coagulase-negative Staphylococcus* (CoNS), spanning 15 species, dominated by *S. saprophyticus*, *S. cohnii* and *S. sciuri*. The only coagulase-positive *Staphylococcus* was *S. aureus* (n = 8) and the only species detected across all wastewater sources. Resistance was highest to benzylpenicillin (88%) and fusidic acid (82%), whereas all isolates remained susceptible to glycopeptides, tigecycline, and linezolid. Fusidic acid resistance was higher in community-wastewater isolates, whereas β-lactam resistance predominated in hospital-wastewater isolates. Sixty percent of CoNS were multidrug-resistant; methicillin resistance occurred in 37.5% of CoNS and 50% of *S. aureus*. Wastewater is a reservoir of diverse multidrug-resistant staphylococci, underscoring One Health relevance reflecting the potential for circulation between humans, animals, and the shared environment. WBS can support population-level AMR monitoring to inform public health and veterinary interventions.

## Simple Summary

Antimicrobial resistance, when bacteria change in ways that make antibiotics no longer effective, has become one of the most serious global healthcare challenges of our time. This study examined bacteria found in wastewater in Dubai to better understand how resistant bacteria spread across the community, hospitals and the environment. The focus was on a group of bacteria called *Staphylococcus*, some of which can cause a wide range of infections in humans and animals, from mild skin infections to serious bloodstream diseases. Over an eight-month period, wastewater samples were collected from community locations, hospitals, and wastewater treatment plants. The bacteria were isolated and tested in the laboratory to determine which antibiotics could stop them and which ones couldn’t. We found a diverse range of *Staphylococcus* species, including several that showed resistance to multiple commonly used antibiotics. Reassuringly, some strong antibiotics remained effective against these bacteria. Overall, our findings suggest that wastewater can act as an important early warning system for detecting and tracking antimicrobial resistance before it becomes a larger public health problem. Monitoring wastewater offers valuable insights into how resistance spreads and can help guide actions to protect humans, animals and the environment.

## 1. Introduction

*Staphylococcus* species are clinically significant pathogens that can cause a wide range of infections, including superficial and deep skin, soft tissue, wound [1], blood stream [2] and device related infections [3] primarily due to their high virulence and their pronounced ability to form biofilms [4]. They are increasingly recognized for their ability to acquire and disseminate antimicrobial resistance (AMR) genes [5]. To date, 94 *Staphylococcus* species have been identified, of which 88 are coagulase-negative *Staphylococcus* (CoNS) [6]. Although CoNS species such as *S. epidermidis* and *S. haemolyticus* were traditionally considered commensals, they are now increasingly being identified as drug-resistant opportunistic pathogens causing infections in humans and animals [7,8]. Coagulase-positive staphylococci (CoPS), such as *S. aureus* and *S. intermedius*, also continue to pose well established risks in the human and veterinary sectors [9]. Assessing both CoNS and CoPS in wastewater enables the investigation of these pathogens, which are of clinical relevance across human and animal sectors, as well as exposes the broader resistance-spread dynamics across shared environments. This is crucial as CoNS species, in particular, are hypothesized to function as major hubs of gene exchange and reservoirs of genetic diversity within the genus [10]. Through mobile genetic elements such as plasmids and bacteriophages, they have the potential to facilitate horizontal gene transfer, thus increasing the risk of emergence of multidrug resistance in pathogenic and non-pathogenic staphylococci.

In addition to the challenge posed to human health, CoPS and CoNS also represent important veterinary pathogens with wide-ranging impact across animal species [4,11]. They are frequently implicated in skin, wound, and intramammary infections in livestock, companion animals, and wildlife [4,11]. In cattle, *S. aureus* is a well-established cause of bovine mastitis, resulting in significant economic losses due to reduced milk yield and increased veterinary expenditures [12]. In horses, species such as *S. xylosus* and *S. sciuri*, are associated with skin and wound infections [13], *S. lentus*, *S. sciuri*, and *S. xylosus* have been isolated from camel skin and nasal flora, suggesting they may act as opportunistic pathogens in camels [14]. Domestic birds, including poultry, may harbor *S. carnosus*, *S. xylosus*, *S. cohnii* and *S. lentus* [15]. In aquaculture, *S. xylosus* infections in rainbow trout have been linked to high mortality and substantial economic losses [16]. In addition, other *Staphylococcus* species have been reported in marine animals, including *S. delphini* in Adélie penguins and *S. pseudintermedius* in Weddell seals [17]. These diverse staphylococcal species, including *S. aureus*, *S. lentus*, and *S. sciuri*, have also been detected in wastewater systems [18,19], highlighting their persistence in shared environments. Collectively, these observations emphasize the interconnectedness of humans, animals, and the environment, reinforcing the need for a One Health approach.

While clinical surveillance remains central to monitoring infectious pathogens, environmental sources, especially wastewater systems, are increasingly being recognized for their role as major reservoirs for resistant pathogens, AMR genes, and mobile genetic elements [20]. Wastewater integrates inputs from domestic, hospital, and livestock settings, providing a composite view of community-level microbial populations, including CoNS and CoPS, as well as their resistance profiles [21]. Within this complex microbial ecosystem, horizontal gene transfer can occur, promoting the exchange of resistance and virulence determinants among diverse bacterial species. Wastewater-based surveillance (WBS) has recently gained momentum as a practical and cost-effective tool for population-level monitoring of pathogens and AMR [22]. Initially widely utilized during the COVID-19 pandemic, WBS has since been applied to track enteric viruses, and AMR genes in various settings [23,24]. Several studies have shown that staphylococcal species can be consistently isolated from municipal, hospital, and industrial wastewater, largely due to their shedding from skin, mucosal surfaces, infected wounds, and contaminated medical waste [18,21,25,26,27,28].

Despite this increasing global interest in WBS for AMR, there remains a paucity of data from the Arabian Gulf region, including the United Arab Emirates (UAE) [29,30]. Although a recent report from the UAE documented the occurrence of staphylococcal species in wastewater [29], systematic data on the diversity and resistance profiles of staphylococcal species in wastewater from various sources, including community and hospital settings is lacking. To address this gap, our study aimed to determine the prevalence, diversity, and AMR profiles of *Staphylococcus* species in wastewater collected from multiple sources, including community, hospital, and wastewater treatment plant (WWTP) sites in Dubai, UAE. We employed a culture-based phenotypic approach to isolate and characterize viable *Staphylococcus* species, thereby supporting the development of WBS as a tool for AMR monitoring in the UAE.

## 2. Materials and Methods

### 2.1. Study Design and Sample Collection

Wastewater samples were collected once every month from June 2024 to January 2025. Samples were obtained from nine community pumping stations, two tertiary healthcare facilities and the two WWTP in the city. Wastewater samples from community sewer nodes and hospital wastewater outlets were collected using the grab sampling method [21] while WWTP influent and effluent samples were collected using 24-h composite sampling [31]. Composite samples were obtained using autosamplers (Hach, Loveland, CO, USA), where samples get automatically accumulated over 24 h in refrigerated containers. During each month, wastewater from all sampling sites was collected within a ±1-day interval to ensure temporal alignment. Between successive months, the sampling date varied by no more than ±2 days, thereby maintaining consistent temporal spacing across the study period. All samples were collected into sterile wide-mouth 1 L bottles (Azlon, Stone, Staffordshire, UK), maintained under a cold chain at 4 °C, and transported to the laboratory. Table 1 shows the sampling strategy, site distribution and frequency that was followed.

### 2.2. Sample Processing and Primary Isolation

Wastewater samples were received at the laboratory and processed immediately. Each wastewater bottle was mixed briefly, and a 15 mL aliquot [32] was transferred into a sterile conical tube (Corning, NY, USA). Samples were centrifuged at 7500 rpm for 10 min, and the supernatant was discarded. The resulting pellet was resuspended directly in 10 mL of Baird-Parker broth with potassium tellurite (HiMedia, Mumbai, India) and incubated on a Stuart orbital shaker (Stuart, Stone, UK) at 37 °C and 125 rpm for 24 h. After selective enrichment, aliquots were streaked onto mannitol salt agar (Medisynal, Dubai, United Arab Emirates) and incubated at 37 °C for 18–24 h. For each sample, plates were examined for macroscopically distinct colony morphotypes, defined by differences in colony form, elevation, margin, size, texture and color [33]. From each plate, up to 5 representative colonies per distinct morphotype were selected for further work. Streaking and subculturing were carried out under aseptic conditions with sterile disposable loops to minimize the risk of contamination. All morphologically distinct colonies were subcultured onto sheep blood agar (Medisynal, Dubai, United Arab Emirates) and incubated at 37 °C for 18–24 h to promote growth. Plates were then inspected for uniform colony morphology and hemolysis patterns; cultures showing mixed growth were re-streaked until pure.

**Figure 1:**
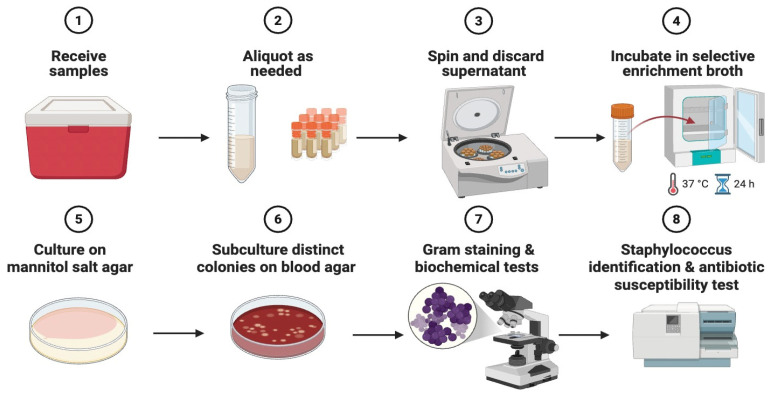
Processing of wastewater samples for detection of *Staphylococcus* species (Created in BioRender https://app.biorender.com/illustrations/68eead61fb09907b6cbc4512 accessed on 27 October 2025).

Representative colonies from each distinct type were subjected to Gram staining and examined microscopically for the presence of Gram-positive cocci (Figure 1). Isolates consistent with staphylococcal species were subjected to biochemical testing (Figure 1), including catalase and coagulase assays. Isolates were preserved in tryptic soy broth (TSB) (Millipore, Darmstadt, Germany) containing 25% glycerol and stored at −80 °C for subsequent analyses.

### 2.3. Species-Level Identification

Presumptive isolates were further confirmed and identified to the species level using the VITEK^®^ 2 compact automated system with Gram Positive identification (ID-GP) cards (bioMérieux, Marcy L’Etoile, France). Bacteria were first cultured on blood agar plates, and fresh overnight growth was used for identification. Pure colonies were suspended in sterile saline to a standardized turbidity of 0.5–0.6 McFarland on DensiCHEK Plus (bioMérieux, Marcy L’Etoile, France) and loaded into the GP cards, which contains a panel of biochemical assays such as carbohydrate utilization and enzyme activities (e.g., urease, arginine dihydrolase). The VITEK^®^ 2 system automatically incubated and monitored metabolic reactions, after which species-level identification was determined.

### 2.4. Antimicrobial Susceptibility Testing (AST)

Bacterial identification, confirmation of MRSA and phenotypic antimicrobial susceptibility testing were performed using the VITEK^®^ 2 Compact automated system with the AST-P592 cards (bioMérieux, France), in accordance with manufacturer-provided protocols and Clinical and Laboratory Standards Institute (CLSI) [34]. Antibiotic susceptibility testing with VITEK^®^ 2 AST cards is based on the broth microdilution; the system automatically incubates and monitors growth in the presence of antimicrobials and uses the Advanced Expert System (AES) to generate MIC values and categorical interpretations. Categorical interpretations were assigned according to the CLSI Performance Standards for Antimicrobial Susceptibility Testing [34]. Multidrug resistance (MDR) was defined as non-susceptibility to at least one agent in three or more classes of antibiotics [35].

### 2.5. Whole Genome Sequencing (WGS)

This was carried out at CosmosID Laboratories (Germantown, MD, USA). Briefly, genomic DNA was extracted from all *S. aureus* wastewater isolates using the DNeasy PowerSoil Pro Kit (Qiagen, Hilden, Germany) according to the manufacturer’s instructions, and DNA was quantified using a Qubit Flex fluorometer (Thermo Fisher Scientific, Waltham, MA, USA). Libraries were prepared with the Watchmaker DNA Library Prep Kit. Sequencing was performed on the Element AVITI platform using 2 × 150 bp paired-end chemistry (Cloudbreak kit; Element Biosciences, San Diego, CA, USA). WGS data are available on NCBI BioProject ID: 1376508.

### 2.6. Bioinformatics and Genomic Analyses

Whole-genome sequencing data from all *S. aureus* isolates were processed using the Bactopia v3.2.0 pipeline [36,37]. Raw paired-end reads underwent quality control and trimming, followed by de novo assembly. Molecular typing included MLST using the *S. aureus* PubMLST scheme, *spa* typing, SCC*mec* typing and core-genome MLST to infer genomic relatedness using RIDOM SeqSphere+ v.10.0.5 EULA (Ridom GmbH, Muenster, Germany), applying a ≤24-allele difference threshold for closely related isolates. AMR genes were identified with AMRFinderPlus v.4.0.19 and Abritamr v.1.0.19.

## 3. Results

### 3.1. Detection of Staphylococcal Species

**Figure 2:**
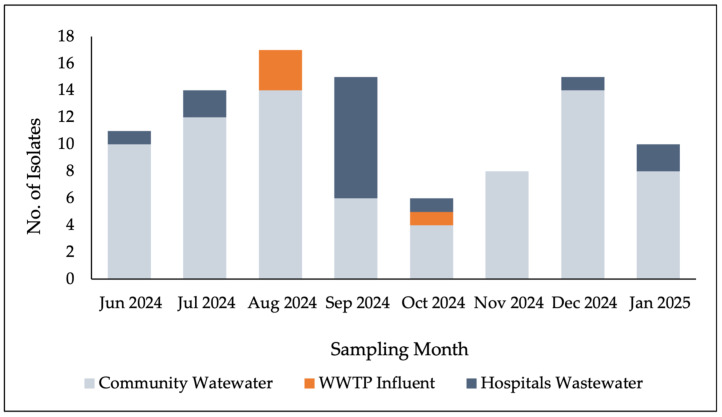
Distribution of isolates from across different wastewater sources.

A total of 96 staphylococcal isolates were recovered from three wastewater sources: hospital wastewater, community wastewater, and WWTP influent. The number of captured isolates per month ranged from 6 to 17, with no statistically significant difference across months (*p* = 0.28). Of these 91.7% (n = 88) were coagulase-negative staphylococci (CoNS) and 8.3% (n = 8) were coagulase-positive *S. aureus* (CoPS), yielding a CoNS:CoPS ratio of approximately 11:1. No staphylococci were detected in WWTP effluent samples. The highest number of isolates were obtained from community wastewater (n = 67), followed by hospital wastewater (n = 16), while only 4 isolates were recovered from WWTP influent (Figure 2).

**Figure 3:**
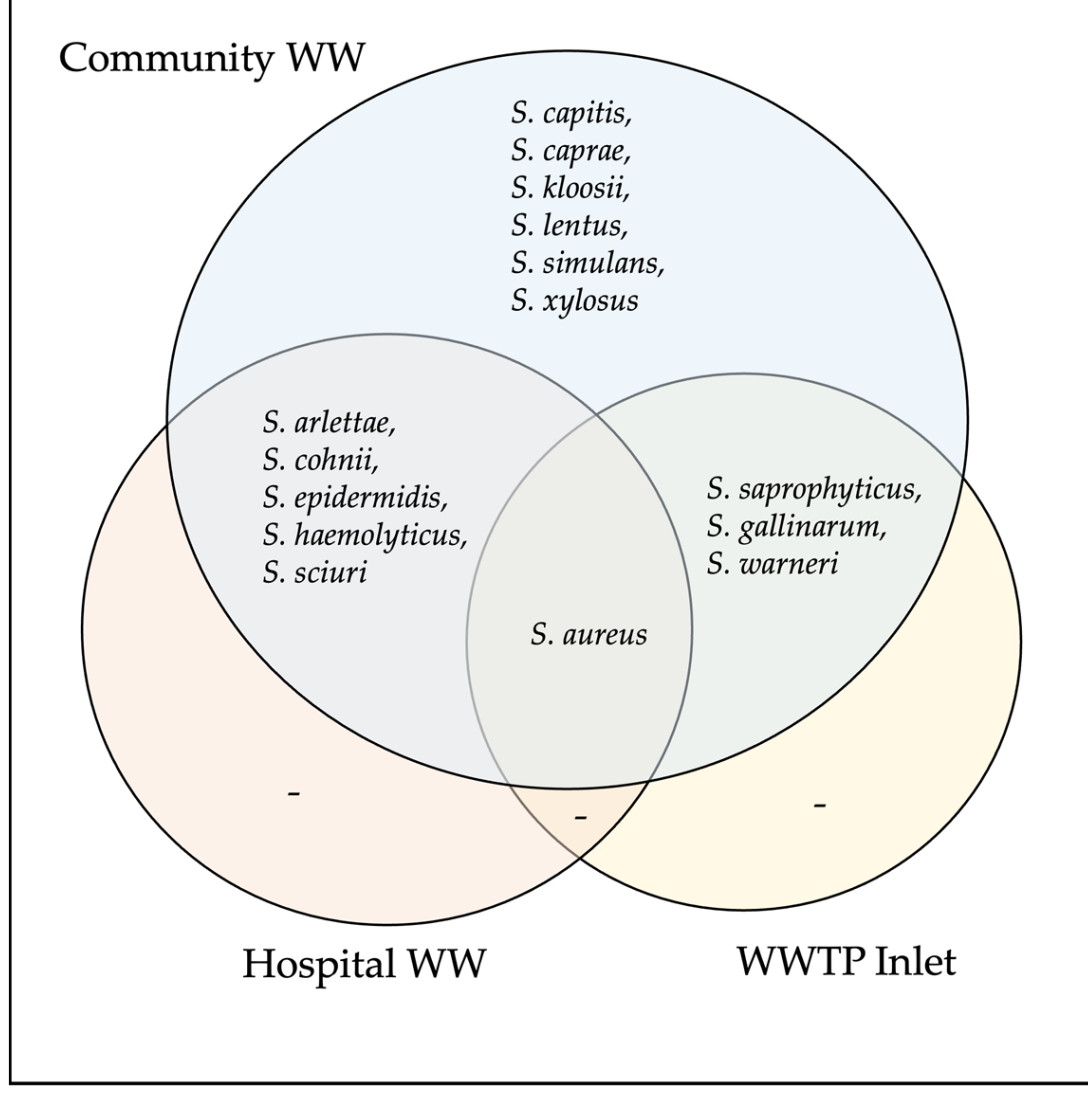
Venn diagram showing the distribution *Staphylococcus* species detected across different wastewater sources.

The 15 *Staphylococcus* species were *S. aureus*, *S. arlettae*, *S. capitis*, *S. caprae*, *S. cohnii*, *S. epidermidis*, *S. gallinarum*, *S. haemolyticus*, *S. kloosii*, *S. lentus* (now *Mammaliicoccus lentus*), *S. saprophyticus*, *S. sciuri* (now *Mammaliicoccus sciuri*), *S. simulans*, *S. warneri*, *and S. xylosus*. The most frequently identified species were *S. cohnii* (n = 15), *S. saprophyticus* (n = 16), *S. sciuri* (n = 13), *S. haemolyticus* (n = 9), and *S. xylosus* (n = 9). The distribution of *Staphylococcus* species across wastewater sources is shown in Figure 3.

### 3.2. Antimicrobial Susceptibility

The majority of the isolates, 88% (n/N = 84/96) and 82% (n/N = 79/96), were resistant to benzylpenicillin and fusidic acid, respectively. Fifty-four isolates were resistant to erythromycin, while resistance to clindamycin and folate inhibitors (trimethoprim/sulfamethoxazole) was detected in 22 and 18 isolates, respectively. All isolates were susceptible to glycopeptides (vancomycin and teicoplanin), as well as to tigecycline and linezolid.

The antibiotic resistance profile of the tested *Staphylococcus* species varied across isolates. On average, each isolate exhibited resistance to approximately four out of the 16 antibiotics on the VITEK panel. However, *S. capitis*, *S. epidermidis* and *S. simulans* isolates showed resistance to only benzylpenicillin. Detailed antibiotic resistance profile for each isolate is shown in Supplementary Table. Resistance to 3 or more classes of antibiotics indicative of MDR phenotype [35] was observed in the majority of staphylococci species identified (n/N = 11/15). Table 2 shows the distribution of MDR isolates across identified species.

Overall, 60% of the CoNS isolates exhibited an MDR phenotype (n = 53), predominantly represented by *S. cohnii* (n = 13) and *S. saprophyticus* (n = 11). As shown in Table 2, MDR isolates were distributed across multiple *Staphylococcus* species, with 33 identified as methicillin-resistant CoNS (MR-CoNS).

Phenotypic methicillin resistance was detected in 33 CoNS and 4 CoPS. The methicillin resistant CoNS include *S. arlettae*, *S. caprae*, *S. cohnii*, *S. epidermidis*, *S. haemolyticus*, *S. saprophyticus*, *S. sciuri* and *S. warneri* isolates. These were detected from community WW, WWTP inlet and hospital WW. Of the 8 *S. aureus* isolates detected, 4 (50%) were methicillin-resistant *S. aureus* (MRSA). These MRSA isolates were from WWTP influent (n = 1) and community wastewater (n = 3).

### 3.3. Whole-Genome Sequencing of S. aureus Isolates

**Figure 4:**
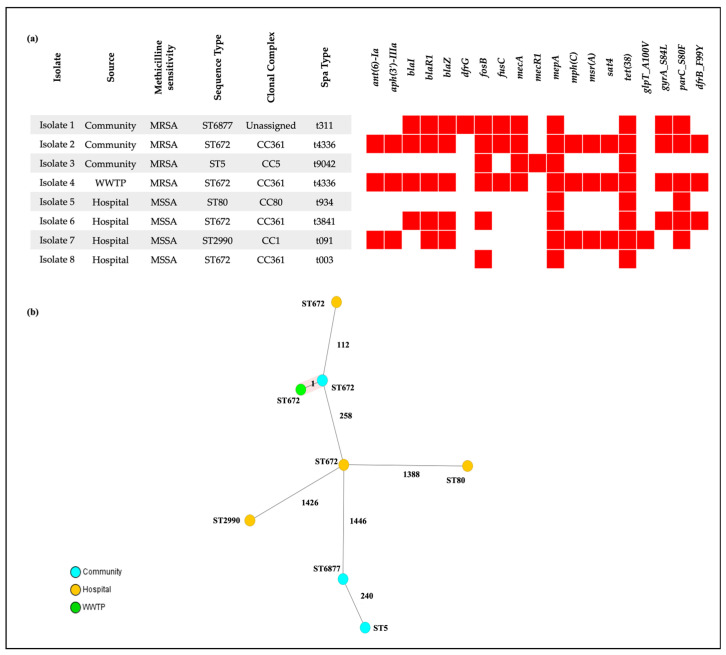
Population structure and antimicrobial resistance genes of *S. aureus* isolates from wastewater. (**a**) Heatmap showing the distribution of AMR genes across eight *S. aureus* genomes, alongside isolate metadata (source, methicillin phenotype, ST, clonal complex, and spa type). Red cells indicate presence of the listed determinant; white cells indicate absence. (**b**) Core genome multilocus sequence typing (cgMLST) comparison of *S. aureus* isolates based on 1861 gene loci. cgMLST minimum-spanning tree of all *S. aureus* isolates (including both MSSA and MRSA) identified in the study.

Whole-genome sequencing of the 8 *S. aureus* isolates revealed that the dominant sequence type (ST) was ST672 (n/N = 4/8; 50%) with the STs identified in MRSA being ST672 (n = 2), ST5 (n = 1), and ST6877 (n = 1). MSSA had isolate each of ST80 and ST2990, and two isolates were ST672. We identified 7 *spa* types with t4336 associated with ST672 detected in two isolates and the remaining *spa* types were each represented by a single isolate (Figure 4a). Two MRSA isolates (ST672 and ST6877) harboured SCC*mec* type V, while the ST5 isolate carried type IV. Phylogenomic analysis showed that the isolates were genetically diverse (Figure 4b). In the cgMLST scheme, all isolate pairs differed by more than 24 alleles, consistent with substantial genomic distance. A single exception was observed: one WWTP influent derived MRSA isolate differed by only one allele from a community derived MRSA isolate (Figure 4b). The MRSA isolates haboured *mecA* gene and largely exhibited a larger repertoire of resistance genes compared to MSSA isolates although the *tet(38)* and *mepA* resistance were universally detected across all isolates (Figure 4a).

## 4. Discussion

This study applied a culture-based approach to investigate antimicrobial resistance in *Staphylococcus* isolated from multiple wastewater sources. Sampling locations were selected to reflect diverse urban activities, including community and hospital wastewater, as well as WWTP influent and effluent, thereby allowing for a comprehensive assessment of isolates from wastewater sources. Our findings align with global reports that demonstrate the occurrence of diverse CoNS and CoPS in various wastewater sources [5,19,21,26,27]. The identification of opportunistic pathogens such as *S. haemolyticus* and *S. cohnii*, alongside the widespread presence of multidrug-resistant isolates, particularly those exhibiting β-lactam and fusidic acid resistance phenotypes, is noteworthy [38,39]. The predominance of these environmental staphylococci, particularly the widespread detection of CoNS species across multiple wastewater sources, suggests substantial ecological resilience. Moreover, the high levels of resistance to major antibiotic classes may reflect selective pressures exerted by residual antibiotics, disinfectants, and heavy metals within wastewater systems [40,41]. Furthermore, several species recovered from wastewater are known to colonize livestock, companion animals, and wildlife [11].

The consistent presence of animal-associated staphylococci, including *S. sciuri* and *S. cohnii*, which were the predominant CoNS identified, suggests that animal-derived contributions influence the wastewater microbiome in our setting. Such overlap between human and animal staphylococcal populations highlights the possibility of interspecies transmission. Wastewater thus provides a One Health interface where microbial and resistance determinants from multiple sectors intersect and where horizontal gene transfer is likely to occur [42,43]. Notably, *S. sciuri*, which is generally considered a non-pathogenic environmental species, has recently been shown to predominate in the farm environment as well as readily adapt to and persist in health care settings [44]. Additionally, *S. sciuri* harboring the *mecA* gene and resistance genes for lincosamide and streptogramin [44], have been reported in the literature, suggesting their potential to serve as reservoirs of AMR genes that may be transferred to pathogenic staphylococci, including *S. aureus*. In this study, there was high occurrence of *S. sciuri*, including isolates that showed methicillin resistance and MDR phenotype. Further work to investigate their genomic profiles, characterize associated AMR genes, and assess potential horizontal gene transfer events is warranted.

The findings demonstrate widespread antibiotic resistance among the staphylococci isolates identified, with the majority (82%) exhibiting resistance to fusidic acid, particularly among CoNS, such as *S. haemolyticus*, *S. cohnii*, and *S. saprophyticus*. These findings provide the first insight into the occurrence of fusidic acid resistance among CoNS and non-clinical CoPS isolates from our setting. In addition, whilst fusidic acid resistance predominated in isolates from community wastewater samples, β-lactam resistance was more frequently observed in isolates from hospital wastewater. This distribution likely reflects the differential antibiotic usage patterns, with β-lactams being more commonly administered in clinical settings compared to topical fusidic acid in community settings. Previous reports from the UAE and the broader Arabian Gulf region have documented a high prevalence of fusidic acid resistance in clinical *S. aureus* isolates, which often carry the SCC*mec*+SCC*fusC* composite resistance genes. Further work is needed to determine the molecular makeup of these environmental CoNS with fusidic acid resistance and explore relationships with clinical *S. aureus* isolates harboring SCC*mec*+SCC*fus* composite genes.

Interestingly, the sole CoPS identified in our study was *S. aureus*, and it was the only species found across all three wastewater sources. Notably, MRSA has been commonly associated with hospital wastewater in reported studies [25,45]. Thus, the detection of MSSA in hospital wastewater, with MRSA being found in community wastewater and WWTP influent, differs from the patterns reported in the literature [25,46]. This detection of multidrug-resistant MRSA in community wastewater and WWTP influent suggests that highly pathogenic strains are circulating more broadly in our setting. Such divergence from reported literature may reflect variations in wastewater inputs, or differences in AMR epidemiology such as the growing prevalence of community-associated MRSA lineages in our setting [47,48]. This underscores the value of regionally tailored WBS studies, as reliance on global patterns risks overlooking locally relevant dynamics.

Molecular characterization of food and clinical *S. aureus* isolates from the UAE has revealed a predominance of community-associated MRSA lineages [47,49], although WGS data for wastewater-derived isolates remain limited. The eight *S. aureus* genomes analysed here therefore provide new insight into this environmental reservoir, demonstrating the presence of genetically distinct MRSA and MSSA isolates. In the cgMLST analysis, all isolate pairs differed by more than 24 alleles, with the exception of two ST672 isolates, indicating an overall lack of close relatedness.

ST672 belongs to CC361, thus making CC361, which has been described as an emerging community-associated lineage [50,51] the predominant CC in this study. Its detection in retail meat products [52], alongside reports from cattle and monkeys [53], and the identification of ST672 in fish and fishery environments [54] have all been documented in the literature, underscoring its relevance across multiple ecosystems. Resistance determinants associated with reduced susceptibility to tetracyclines and fluoroquinolones were present in all isolates, raising concern for treatment options should animals or humans be exposed to such strains. Furthermore, the identification of plasmid replicons *rep16* and *rep5a* suggests a potential for horizontal gene exchange by these isolates.

In the UAE, treated wastewater effluent from WWTPs is commonly reused for irrigation due to limited freshwater resources. Staphylococci were not detected in any WWTP effluent, which suggests that the wastewater treatment likely removed viable cells, although the presence of viable but non-culturable Staphylococci in WWTP effluent remains a possibility. In addition, staphylococci or their associated AMR determinants may have partitioned into the sludge fraction, which was not assessed in this study. As sewage sludge is frequently applied to agricultural land as fertilizer, this represents a potential transmission route that merits further investigation. Moreover, resistance genes may persist in treated effluent and, when reused for irrigation, could re-enter the environment, expanding the reservoir of resistant staphylococci, promoting horizontal gene transfer to other bacteria, and increasing the potential for human and animal exposure through contaminated soil, water, or agricultural produce. Future work employing shotgun metagenomic sequencing is recommended to identify and determine the abundance of AMR genes and mobile genetic elements in WWTP effluent.

Culture-based approaches remain a critical component of wastewater surveillance, enabling the isolation of viable bacteria for antimicrobial susceptibility testing and thereby revealing resistant phenotypes of clinical and veterinary relevance. As wastewater contains a complex and often low-abundance microbial community, culture methods also facilitate the enrichment of rare organisms, including staphylococcal species that may be underrepresented or missed entirely by metagenomic analyses. Importantly, cultured isolates provide isolates for downstream bacterial whole-genome sequencing work. However, applying culture-based methods to wastewater is inherently challenging. Competition from diverse microbial populations and exposure to environmental stressors, such as residual antibiotics, biocides, toxic compounds, and lytic phages, can inhibit the growth of more fastidious or low-abundance taxa [43,55,56,57]. In addition, some staphylococcal species may enter a viable but non-culturable state, in which cells remain metabolically active and retain their resistance determinants but fail to grow on routine laboratory media. Differences in isolate recovery between community sites and WWTP samples suggest that environmental pressures in centralized systems may impact culturability, underscoring the value of sampling across multiple wastewater sources.

## 5. Conclusions

In conclusion, this study demonstrates the utility of culture-based methods for detecting viable staphylococci across multiple wastewater sources. While wastewater treatment reduces the prevalence of staphylococci, the consistent detection of MDR isolates in upstream sources underscores the role of wastewater as a reservoir and potential convergence for both human- and animal-associated microbes. Within a One Health framework, these findings highlight cross-sector transmission risks and provide a foundation for implementing AMR WBS to protect human and animal health.

## Figures and Tables

**Table 1:** Overview of wastewater sampling strategy (June 2024–January 2025).

Parameter	Nine Community Wastewater (Pumping Stations)	Two Hospital Wastewater (Pre-Sewer Junction)	Two Wastewater Treatment Plants (Influent & Effluent)
Sampling Method	Grab	Grab	24-h Composite
No. of Samples	72	16	32
Total No. of Samples	120 wastewater samples		

**Table 2:** Distribution of multidrug-resistant isolates identified for each *Staphylococcus* species.

*Staphylococcus* Species	*S. arlettae*	*S. aureus*	*S. capitis*	*S. caprae*	*S. cohnii*	*S. epidermidis*	*S. gallinarum*	*S. haemolyticus*	*S. kloosii*	*S. lentus*	*S. saprophyticus*	*S. sciuri*	*S. simulans*	*S. warneri*	*S. xylosus*
Total number of isolates detected	6	8	1	1	15	6	5	9	2	1	16	13	1	3	9
No. of multidrug-resistant * isolates	6	3	0	1	13	4	0	7	2	0	11	4	0	3	2
No. of methicillin-resistant isolates	1	4	0	1	10	3	0	7	2	0	6	1	0	2	0
No. of penicillin-resistant isolates	6	7	1	1	15	6	5	8	2	0	16	5	1	3	8

* Resistance to ≥3 antibiotic classes [35].

## Data Availability

The original contributions presented in this study are included in the article/Supplementary Material. Further inquiries can be directed to the corresponding author.

## Supplementary Materials

The following supporting information can be downloaded at: https://www.mdpi.com/article/10.3390/vetsci13010014/s1, Table S1: *Staphylococcus* isolates recovered from wastewater samples collected between June 2024 and January 2025, with their antibiotic susceptibility profiles.

## Abbreviations

The following abbreviations are used in this manuscript:

AMR	Anti-microbial Resistance
AST	Antimicrobial Susceptibility Testing
cgMLST	Core genome multilocus sequence typing
CoNS	Coagulase-negative staphylococci
CoNs	Coagulase-positive staphylococci
CoPS	Coagulase-positive staphylococci
HGT	Horizontal Gene Transfer
ID-GP	Identification—Gram-Positive
MDR	Multidrug-resistant
MICs	Minimum Inhibitory Concentrations
MR-CoNS	Methicillin-resistant coagulase-negative staphylococci
MRSA	Methicillin-resistant *Staphylococcus aureus*
MSSA	Methicillin-susceptible *Staphylococcus aureus*
TSB	Tryptic Soy Broth
UAE	United Arab Emirates
WBS	Wastewater-Based Surveillance
WGS	Whole Genome Sequencing
WWTP	Wastewater Treatment Plant
